# A new monoclinic polymorph of *trans*-dichloridodipyridine­palladium(II)

**DOI:** 10.1107/S1600536808033552

**Published:** 2008-10-22

**Authors:** Hon Man Lee, Chuang-Yi Liao

**Affiliations:** a National Changhua University of Education, Department of Chemistry, Changhua 50058, Taiwan

## Abstract

In the structure of the title compound, [PdCl_2_(C_5_H_5_N)_2_], the Pd^II^ atom is located on an inversion centre and the pyridine rings are coplanar. There is inter­molecular π–π stacking between the pyridyl rings, with a centroid-to-centroid separation of 3.916 (1) Å. The structure is a new polymorph of two previously determined structures [Viossat, Dung & Robert (1993 ▶). *Acta Cryst.* C**49**, 84–85; Liao & Lee (2006 ▶). *Acta Cryst.* E**62**, m680–m681].

## Related literature

For the other two polymorphs of the title compound, see: Viossat *et al.* (1993 ▶); Liao & Lee (2006 ▶).
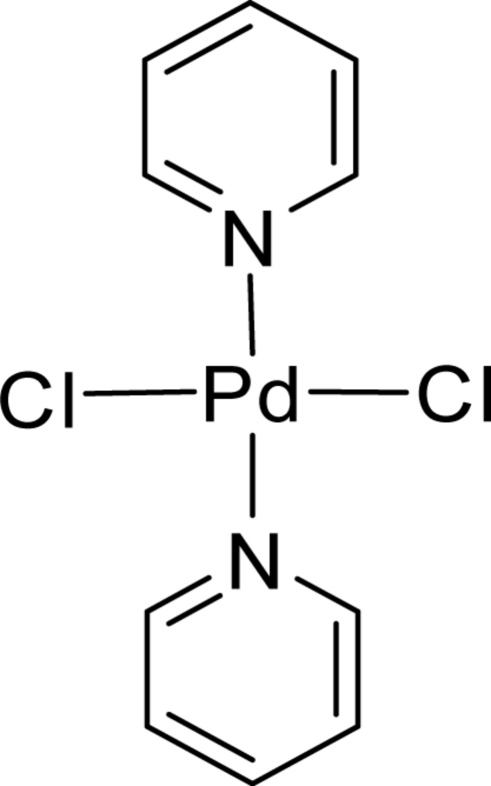


## Experimental

### 

#### Crystal data

[PdCl_2_(C_5_H_5_N)_2_]
*M* *_r_* = 335.50
Monoclinic, 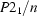

*a* = 3.9159 (2) Å
*b* = 8.7921 (4) Å
*c* = 16.2974 (8) Å
β = 90.442 (3)°
*V* = 561.09 (5) Å^3^
*Z* = 2
Mo *K*α radiation
μ = 2.10 mm^−1^
*T* = 150 (2) K
0.45 × 0.10 × 0.07 mm

#### Data collection

Bruker SMART APEXII diffractometer
Absorption correction: multi-scan (*SADABS*; Sheldrick, 2003 ▶) *T*_min_ = 0.452, *T*_max_ = 0.867
5861 measured reflections
1445 independent reflections
1314 reflections with *I* > 2σ
*R*_int_ = 0.028

#### Refinement

*R*[*F* ^2^ > 2σ(*F* ^2^)] = 0.023
*wR*(*F* ^2^) = 0.056
*S* = 1.10
1445 reflections
70 parameters
H-atom parameters constrained
Δρ_max_ = 0.91 e Å^−3^
Δρ_min_ = −1.03 e Å^−3^

### 

Data collection: *APEX2* (Bruker, 2004 ▶); cell refinement: *SAINT* (Bruker, 2004 ▶); data reduction: *SAINT*; program(s) used to solve structure: *SHELXTL* (Sheldrick, 2008 ▶); program(s) used to refine structure: *SHELXTL*; molecular graphics: *SHELXTL*; software used to prepare material for publication: *SHELXTL*.

## Supplementary Material

Crystal structure: contains datablocks I, global. DOI: 10.1107/S1600536808033552/bi2308sup1.cif

Structure factors: contains datablocks I. DOI: 10.1107/S1600536808033552/bi2308Isup2.hkl

Additional supplementary materials:  crystallographic information; 3D view; checkCIF report

## Figures and Tables

**Fig. 1.**
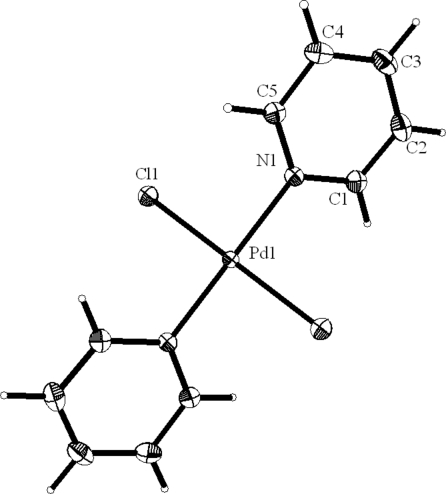
Molecular structure of the title compound, showing 50% displacement ellipsoids for non-H atoms. The H atoms are dipicted by circles of an arbitrary radius. The unlabelled atoms are related to the labelled ones by -x, 1 - y, -z.

**Fig. 2.**
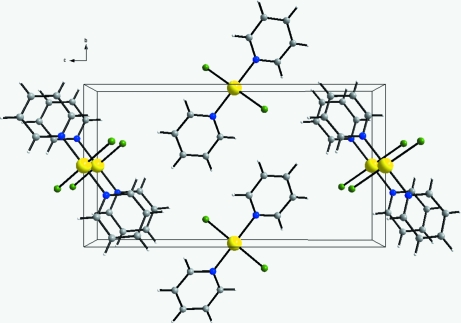
Packing diagram of the title compound viewed along the a axis.

## supplementary crystallographic information

### Comment

Two polymorphic forms of the title compound have already been determined (Viossat *et al.*, 1993; Liao & Lee, 2006). The polymorphic form determined by us previously has a plate-like crystal habit (Liao & Lee, 2006). Herein, we present a new polymorphic form of the title compound. This new form has a rod-like habit. The Pd^II^ atom, situated at a centre of inversion, has a square-planer coordination geometry with two *trans* pyridine ligands and two *trans* chloride ligands (Fig. 1). Similar to the polymorph determined by us previously (Liao & Lee, 2006), in this new polymorphic form the two pyridine rings are co-planar. The co-planariity in these two forms is in sharp contrast to that in the other polymorph in which the the two pyridine planes make an angle of 160.0 (5)° (Viossat *et al.*, 1993).

The crystal packing is distinctly different in the three polymorphs. A view of the packing arrangement for the new polymorphic form is shown in Fig. 2. Intermolecular π–π stacking exists between the pyridyl rings, with centroid–centroid separation 3.916 Å.

### Experimental

The title compound is commercially available. Crystals were grown by slow diffusion of diethyl ether into a dimethylformamide solution containing the compound. The polymorphic form has a rod-like crystal habit.

### Refinement

All H atoms could be identified in the difference Fourier map, but were positioned geometrically and refined as riding atoms, with C—H = 0.95 Å and *U*_iso_(H) = 1.2*U*_eq_(C).

### Crystal data

**Table:**

[PdCl_2_(C_5_H_5_N)_2_]	*F*(000) = 328
*M**_r_* = 335.50	*D*_x_ = 1.986 Mg m^−^^3^
Monoclinic, *P*2_1_/*n*	Mo *K*α radiation, λ = 0.71073 Å
Hall symbol: -P 2yn	Cell parameters from 4043 reflections
*a* = 3.9159 (2) Å	θ = 2.5–34.2°
*b* = 8.7921 (4) Å	µ = 2.10 mm^−^^1^
*c* = 16.2974 (8) Å	*T* = 150 K
β = 90.442 (3)°	Rod, colourless
*V* = 561.09 (5) Å^3^	0.45 × 0.10 × 0.07 mm
*Z* = 2	

### Data collection

**Table:**

Bruker SMART APEXII diffractometer	1445 independent reflections
Radiation source: fine-focus sealed tube	1314 reflections with *I* > 2σ
graphite	*R*_int_ = 0.028
ω scans	θ_max_ = 28.7°, θ_min_ = 2.6°
Absorption correction: multi-scan (SADABS; Sheldrick, 2003)	*h* = −5→5
*T*_min_ = 0.452, *T*_max_ = 0.867	*k* = −11→8
5861 measured reflections	*l* = −22→19

### Refinement

**Table:**

Refinement on *F*^2^	Primary atom site location: structure-invariant direct methods
Least-squares matrix: full	Secondary atom site location: difference Fourier map
*R*[*F*^2^ > 2σ(*F*^2^)] = 0.023	Hydrogen site location: inferred from neighbouring sites
*wR*(*F*^2^) = 0.056	H-atom parameters constrained
*S* = 1.10	*w* = 1/[σ^2^(*F*_o_^2^) + (0.0105*P*)^2^ + 1.3011*P*] where *P* = (*F*_o_^2^ + 2*F*_c_^2^)/3
1445 reflections	(Δ/σ)_max_ < 0.001
70 parameters	Δρ_max_ = 0.91 e Å^−^^3^
0 restraints	Δρ_min_ = −1.03 e Å^−^^3^

### Special details

**Table:**

Geometry. All e.s.d.'s (except the e.s.d. in the dihedral angle between two l.s. planes) are estimated using the full covariance matrix. The cell e.s.d.'s are taken into account individually in the estimation of e.s.d.'s in distances, angles and torsion angles; correlations between e.s.d.'s in cell parameters are only used when they are defined by crystal symmetry. An approximate (isotropic) treatment of cell e.s.d.'s is used for estimating e.s.d.'s involving l.s. planes.
Refinement. Refinement of *F*^2^ against ALL reflections. The weighted *R*-factor *wR* and goodness of fit *S* are based on *F*^2^, conventional *R*-factors *R* are based on *F*, with *F* set to zero for negative *F*^2^. The threshold expression of *F*^2^ > σ(*F*^2^) is used only for calculating *R*-factors(gt) *etc*. and is not relevant to the choice of reflections for refinement. *R*-factors based on *F*^2^ are statistically about twice as large as those based on *F*, and *R*- factors based on ALL data will be even larger.

### Fractional atomic coordinates and isotropic or equivalent isotropic displacement parameters (Å^2^)

**Table:**

	*x*	*y*	*z*	*U*_iso_*/*U*_eq_	
C1	0.1177 (6)	0.6747 (3)	0.15154 (15)	0.0203 (5)	
H1	0.2253	0.5833	0.1690	0.024*	
C2	0.1019 (7)	0.7956 (3)	0.20522 (16)	0.0249 (5)	
H2	0.1976	0.7878	0.2588	0.030*	
C3	−0.0560 (7)	0.9288 (3)	0.17976 (17)	0.0251 (5)	
H3	−0.0737	1.0130	0.2160	0.030*	
C4	−0.1875 (7)	0.9376 (3)	0.10081 (18)	0.0238 (5)	
H4	−0.2913	1.0288	0.0817	0.029*	
C5	−0.1655 (6)	0.8117 (3)	0.05015 (15)	0.0194 (5)	
H5	−0.2595	0.8169	−0.0037	0.023*	
Cl1	0.24377 (16)	0.64576 (7)	−0.10193 (4)	0.01983 (13)	
N1	−0.0149 (5)	0.6822 (2)	0.07511 (12)	0.0159 (4)	
Pd1	0.0000	0.5000	0.0000	0.01359 (8)	

### Atomic displacement parameters (Å^2^)

**Table:**

	*U*^11^	*U*^22^	*U*^33^	*U*^12^	*U*^13^	*U*^23^
C1	0.0236 (11)	0.0198 (12)	0.0174 (11)	0.0000 (9)	−0.0019 (9)	−0.0002 (9)
C2	0.0263 (12)	0.0314 (14)	0.0171 (12)	−0.0021 (11)	−0.0009 (10)	−0.0051 (10)
C3	0.0272 (13)	0.0227 (13)	0.0256 (13)	−0.0032 (11)	0.0024 (10)	−0.0096 (10)
C4	0.0252 (12)	0.0163 (12)	0.0298 (14)	0.0018 (10)	0.0006 (10)	−0.0020 (10)
C5	0.0232 (11)	0.0170 (11)	0.0180 (11)	−0.0001 (9)	−0.0011 (9)	0.0003 (9)
Cl1	0.0255 (3)	0.0174 (3)	0.0167 (3)	−0.0023 (2)	0.0026 (2)	0.0009 (2)
N1	0.0199 (9)	0.0140 (9)	0.0138 (9)	−0.0011 (7)	0.0000 (7)	−0.0012 (7)
Pd1	0.01822 (13)	0.01113 (12)	0.01142 (12)	0.00025 (9)	−0.00070 (8)	−0.00068 (8)

### Geometric parameters (Å, °)

**Table:**

C1—N1	1.347 (3)	C4—H4	0.950
C1—C2	1.379 (4)	C5—N1	1.344 (3)
C1—H1	0.950	C5—H5	0.950
C2—C3	1.386 (4)	Cl1—Pd1	2.3104 (6)
C2—H2	0.950	N1—Pd1	2.017 (2)
C3—C4	1.385 (4)	Pd1—N1^i^	2.017 (2)
C3—H3	0.950	Pd1—Cl1^i^	2.3104 (6)
C4—C5	1.384 (4)		
			
N1—C1—C2	122.0 (2)	N1—C5—C4	121.8 (2)
N1—C1—H1	119.0	N1—C5—H5	119.1
C2—C1—H1	119.0	C4—C5—H5	119.1
C1—C2—C3	118.9 (2)	C5—N1—C1	119.1 (2)
C1—C2—H2	120.5	C5—N1—Pd1	120.25 (16)
C3—C2—H2	120.5	C1—N1—Pd1	120.64 (17)
C4—C3—C2	119.2 (2)	N1^i^—Pd1—N1	180.0
C4—C3—H3	120.4	N1^i^—Pd1—Cl1	89.43 (6)
C2—C3—H3	120.4	N1—Pd1—Cl1	90.57 (6)
C5—C4—C3	119.0 (2)	N1^i^—Pd1—Cl1^i^	90.57 (6)
C5—C4—H4	120.5	N1—Pd1—Cl1^i^	89.42 (6)
C3—C4—H4	120.5	Cl1—Pd1—Cl1^i^	180.0

Symmetry codes: (i) −*x*, −*y*+1, −*z*.
